# Psychosocial factors associated with fatigue in mining work

**DOI:** 10.47626/1679-4435-2025-1492

**Published:** 2025-09-25

**Authors:** Amanda Sorce Moreira, Sergio Roberto de Lucca

**Affiliations:** 1 Programa de Pós-Graduação em Saúde Coletiva, Universidade Estadual de Campinas, Campinas, SP, Brazil

**Keywords:** fatigue, work, mining., fadiga, trabalho, mineração.

## Abstract

**Introduction:**

Fatigue is related to lifestyle habits and working conditions. In the corporate environment, the organization and type of work, as well as social support, can cause or aggravate fatigue and its dimensions (sleepiness and indisposition to work, difficulty concentrating and paying attention, and physical discomfort).

**Objectives:**

To assess the prevalence of fatigue and its association with biosocial characteristics, job demands, and the risk of fatigue in heavy vehicle drivers in the mining sector.

**Methods:**

A cross-sectional study with 111 workers used the biosocial and organizational questionnaire, the Job Stress Scale for psychosocial assessment, and the Yoshitake Fatigue Assessment Questionnaire as instruments.

**Results:**

Fatigue was associated with marital status, education, lifestyle habits, and psychosocial factors at work. Most fatigued workers were exposed to passive work with low social support.

**Conclusions:**

Driving heavy vehicles did not increase the risk of fatigue; however, high work demands and low social support were the psychosocial factors most related to the dimensions of fatigue.

## Abstract (pt)

**Introdução:**

A fadiga está relacionada aos hábitos de vida e às condições de trabalho. No ambiente corporativo, a organização, o tipo de trabalho e o apoio social podem causar ou agravar a fadiga e suas dimensões (sonolência e indisposição para o trabalho, dificuldade de concentração e atenção e ao desconforto físico).

**Objetivos:**

Avaliar a prevalência da fadiga e sua associação com as características biossociais, as exigências do trabalho e o risco de fadiga em motoristas de veículos pesados no setor de mineração.

**Métodos:**

Estudo transversal com 111 trabalhadores, que utilizou como instrumentos o questionário biossocial e organizacional, o Job Stress Scale de avaliação psicossocial e o Questionário de Avaliação da Fadiga de Yoshitake.

**Resultados:**

A fadiga apresentou associação com estado civil, escolaridade, hábitos de vida e fatores psicossociais no trabalho, além de a maioria dos trabalhadores fadigados estarem expostos ao trabalho passivo com baixo apoio social.

**Conclusões:**

Conduzir veículos pesados não aumentou o risco de fadiga, entretanto, as elevadas demandas de trabalho e o baixo apoio social foram os fatores psicossociais que mais se relacionaram com as dimensões da fadiga.

## INTRODUCTION

The contemporary world of work demands high levels of performance and productivity from workers, which can lead to physical, mental, and emotional fatigue. Fatigue syndrome, also known as neurasthenia, is characterized as a psychological disorder resulting from physical and mental fatigue, associated with a certain decline in professional performance and capacity, along with sensations of physical weakness, feelings of exhaustion, bodily pain, and sleep disturbances.^1,2^

Fatigue may be associated with exposure to chemicals during work activities and with psychosocial factors in the workplace, particularly long working hours, intense work pace, shift work and its variations, person-task interaction, and the nature of interpersonal relationships at work.^1^ In addition, individual factors such as age, family problems, and sleep and rest conditions can also increase the likelihood of fatigue and exacerbate its wear-related symptoms.^3^

Fatigue, as both a symptom and indicator of strain, is more prevalent in certain occupational activities and sectors, and its effects pose risks to the health and safety of both workers and service users, notably in the health care, transportation, and aviation sectors. In transportation activities within the mining sector, fatigue has been linked to night work and sleepiness, which are further worsened by domestic chores, responsibilities, and family conflicts.^4^

Jobs involving high physical and cognitive demands increase stress and trigger physiological changes, such as elevated cortisol production and reduced dopamine levels. This leads to fatigue, which is characterized as a subjective feeling of tiredness and discomfort, decreased alertness, attention, concentration, and motivation, psychological disturbances, impaired decision-making ability, daytime sleepiness, muscle loss, and, in more severe cases, the development of chronic fatigue syndrome.^3^

Fatigue symptoms can occur at any point during work, even before it begins, and may impact workers’ motivation and job satisfaction, induce errors, and increase the risk of occupational accidents and work-related illnesses.^5^

Activities involving long working hours, repetitive or monotonous tasks, shift and night work, insecure working conditions, and frequent schedule changes are identified as stressors and contributors to fatigue among professional heavy vehicle drivers.^6^

Fatigue in mining work - particularly among heavy vehicle drivers - is also frequently linked to poor sleep and rest conditions and to the psychological characteristics of the job. Thus, the present study aimed to test the following hypotheses: (1) driving heavy vehicles increases the risk of fatigue; (2) work shift is related to fatigue; (3) lack of sleep and rest increases the risk of fatigue; and (4) the level of job demand and autonomy in mining work may increase the risk of fatigue.

Therefore, this study aimed to assess the prevalence of fatigue and its association with biosocial characteristics, psychosocial work factors, and the risk of fatigue among heavy vehicle drivers in the mining sector, according to levels of job demand and autonomy.

## METHODS

This is an epidemiological, cross-sectional, descriptive study with a quantitative approach, conducted with a sample of workers from a mining company located in Poços de Caldas, in the state of Minas Gerais, Brazil, between September and November 2023.

At the time, the refining and production sectors operated on uninterrupted rotating shifts, while the other departments worked on fixed shifts from Monday to Friday, with rare exceptions on Saturdays and holidays. Work shifts began between 5:00 a.m. and 7:00 a.m. and ended between 3:00 p.m. and 5:00 p.m., depending on the department. Additionally, during the fieldwork period, the company was completing the construction of its first filtration system and employed civil construction workers who operated on rotating daytime shifts (from 6:40 a.m. to 5:15 p.m.) every day of the week.

Sampling was non-probabilistic and based on convenience. The company’s occupational health service provided a list of 65 eligible workers, based on the type of vehicle operated, work schedule, and daily working hours.

Participants were included in the study if they had been routinely operating any type of heavy vehicle (articulated trucks, tractors or agricultural machinery, buses, or minibuses) for more than 90 days. For comparison purposes and to better understand the factors associated with fatigue, a second group of 65 workers who did not operate heavy vehicles - but worked in the same departments as the eligible drivers (mining, factory, and civil construction) - was also invited to participate.

Workers were excluded from the study if they had medical restrictions, were on medical leave or vacation during the study period. Additionally, 12 bus or minibus drivers were excluded because their schedules and work hours differed significantly from the rest of the workforce (they only transported workers at the beginning and end of shifts, totaling approximately 5 hours of work and 3 hours of rest). The final sample consisted of 111 workers.

Data were collected using a printed, self-administered instrument composed of 3 questionnaires: the biosocial and organizational questionnaire, the Job Stress Scale (JSS), and the Yoshitake Fatigue Assessment Questionnaire.

The biosocial and organizational questionnaire was developed by the authors and consisted of 48 mixed questions (multiple choice and semi-open), primarily about lifestyle habits, sleep and rest, and characteristics and perceptions of their work.

The JSS, or “Swedish Demand-Control-Social Support Model,” is a 3-dimensional scale internationally used to assess work-related stress risk factors based on psychosocial aspects. It also classifies work according to the levels of psychological demand and autonomy (control). This study used the nationally validated short version, which contains 17 Likert-type questions: 5 on psychological demand, 6 on task control, and 6 on social support.^7^

The Yoshitake Fatigue Assessment Questionnaire is a highly reliable, self-administered instrument that has been translated and widely used in Brazil by occupational health researchers.^8,9^ It includes 30 multiple-choice questions: 10 related to sleepiness and unwillingness to work, 10 related to concentration and attention difficulties, and 10 related to physical discomfort associated with fatigue. This instrument enables assessment of fatigue across multiple dimensions.^8^

The study was conducted by the researchers with the support of 2 nursing technicians from the institution’s occupational health clinic. At the start of each work shift, researchers provided a brief explanation of the study and invited the target population to participate. Those who agreed signed the informed consent form and, in a separate step, received the questionnaire, which ensured anonymity.

To avoid disrupting production, the questionnaires were completed during work hours, in available rest periods. They were returned in sealed opaque envelopes to the nursing technicians on the same day and then forwarded to the researchers.

All ethical principles for research involving human participants were followed. The study was reviewed and approved by the local Research Ethics Committee, under protocol no. 67697623.6.0000.540, on May 31, 2023.

The JSS analysis was based on the scores of its dimensions. For the demand and control dimensions, the response options were “often,” “sometimes,” “rarely,” and “never or almost never,” with scores ranging from 0 to 4, increasing with the frequency of the event. For the social support dimension, the response options were “strongly agree,” “agree more than disagree,” “disagree more than agree,” and “strongly disagree,” with scores from 0 to 4, increasing with the level of agreement. Scores were totaled, medians calculated, and each dimension was classified as either low or high. To categorize work conditions as high strain, low strain, passive work, or active work, only the median values of the demand and control dimensions were used.

In the Yoshitake Fatigue Questionnaire, each question has 5 answer options, which were converted into scores: “always” was scored as 5, “often” as 4, “sometimes” as 3, “rarely” as 2, and “never” as 1. The total score was calculated, and the median was used to classify fatigue as either low or high.

Data were analyzed using the SAS software, version 9.4, through descriptive statistics and presented as frequency tables with absolute values (n) and percentages (%). Chi-square or Fisher’s exact tests were applied to examine the association between independent variables and the outcome of fatigue and each of its dimensions. Odds ratios (OR) with 95% CIs were also estimated for all variables showing statistically significant associations with fatigue and its dimensions. The significance level adopted for the study was 5%.

## RESULTS

This study revealed a predominance of male workers (84.7%), most of whom were between 30 and 49 years old (45.9%), married or in a stable union (57.7%), and had completed up to secondary education (69.4%). There was no difference between participants with (49.5%) and without dependents (50.5%).

Most participants reported being non-smokers (84.7%), non-drinkers (51.3%), not using sleep-inducing substances (eg, melatonin, herbal teas, or medications) (88.3%), and not consuming energy drinks or stimulants during workdays (77.5%).

Regarding occupational characteristics, most workers were employed by outsourced companies (77.5%) and worked in the civil construction sector (42.4%), on daytime shifts following a 5×2 schedule (85.6%) with 8-hour workdays (53.1%). Most participants had been in their current position for 1 to 4 years (54.9%), operated heavy vehicles (44.1%) for an average of 5 hours without rest (50.5%), and regularly worked at least 2 hours of overtime per week (64%).

Most workers had a commute of less than 1 hour (71.2%), did not have a second job (92.8%), and had never taken a medical leave of absence (79.3%).

Regarding sleep and rest conditions, 36.9% reported having 10 to 11 hours of rest between work shifts, and 61.3% slept an average of 6 to 7 hours per day. Most stated that work did not interfere with sleep quality (90.1%) and rated their sleep quality as “good” (61.2%). Additionally, 86.4% considered their sleep duration sufficient for recovery, and 77.5% reported feeling fully rested upon waking.

As for rest breaks during the workday, most workers stated that they never napped (75.7%), and about one-third (32.4%) remained inside their vehicles during these breaks.

Most workers were satisfied with their wages and benefits (69.3%), training opportunities (69.4%), safety conditions (72.1%), physical infrastructure (73.9%), work equipment (69.4%), and psychosocial aspects of work, such as their tasks and job functions (77.5%), career development opportunities (72.1%), support from supervisors (72.8%) and coworkers (70.8%), and especially from their families (96.4%).

Regarding fatigue, most participants reported low levels of sleepiness and unwillingness to work (54.1%), low difficulty with concentration and attention (56.8%), and low physical discomfort (61.3%). The overall prevalence of high fatigue among workers was 49.5%.

After analyzing and classifying the fatigue questionnaire scores, 55 workers were identified as having high fatigue. Of these, 69.1% were employed by outsourced companies, worked in internal sectors of the mineral processing plant (41.8%), during daytime hours (83.6%), on a 5×2 schedule (81.8%), with 8-hour shifts (54.5%), had been in their current role for 1 to 4 years (54.5%), did not operate heavy vehicles (47.3%), worked at least 2 hours of overtime per week (52.7%), had a commute of less than 1 hour (67.3%) using company-provided transport or chartered buses (54.5%), had no second job (94.5%), and had never taken medical leave (80%).

Regarding sleep and rest conditions among workers with high fatigue, most slept at their own residence during workdays (85.5%), with sleep duration between 6 and 7 hours (60%), felt fully rested upon waking (63.6%), rated their sleep quality in recent months as “good” (69.1%), and reported that work did not interfere with their sleep quality (85.5%) - although it did affect their personal lives (56.4%). These workers also reported not taking naps during rest breaks (72.7%), with 27.3% staying inside a vehicle and 20% in a company rest room during these periods. Overall, even among those with high fatigue, the workers reported being satisfied with all work-related aspects and the support they received from their families (97.3%).

**Figure 1:**
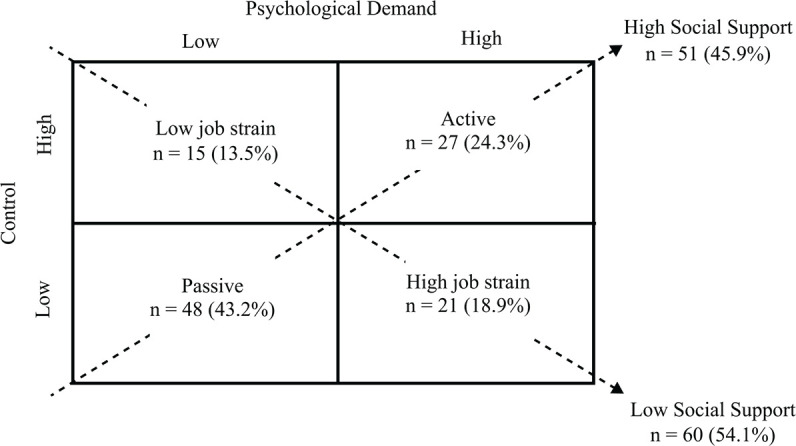
Distribution of workers (n = 111) according to the demand-control-social support model, Poços de Caldas, Minas Gerais, Brazil, 2023.

Work-related stress was evaluated based on the dimensions of psychological demands, control over the work process, and social support, which allowed for the classification of participants into 4 job categories, as displayed in Figure 1.

Descriptive analysis of the scales used to assess work-related stress and fatigue showed that most workers were exposed to low psychological demands (56.7%), low control (62.2%), and low social support (54.1%).

As shown in Table 1, most workers with low demand reported low levels of sleepiness and unwillingness to work (66.7%), low difficulty with concentration and attention (65.1%), and low physical discomfort (69.8%). However, they still exhibited high overall fatigue (46%). Workers with low control reported low sleepiness and unwillingness to work (60.9%), low difficulty with concentration and attention (65.2%), low physical discomfort (62.3%), and low fatigue (56.5%). Similarly, those with low social support also showed low sleepiness and unwillingness to work (60%), low difficulty with concentration and attention (66.7%), low discomfort (63.3%), and low fatigue (63.3%).

**Table 1:** Distribution of workers (n = 111) according to dimensions of stress and fatigue assessment scales, Poços de Caldas, Minas Gerais, Brazil, 2023

Dimension	Cutoff points	n	(%)
Sleepiness and unwillingness to work			
Low	10 to 14	60	54.1
High	15 to 36	51	45.9
Difficulty with concentration and attention			
Low	10 to 17	63	56.8
High	18 to 41	48	43.2
Physical discomfort			
Low	10 to 12	68	61.3
High	13 to 30	43	38.7
Overall fatigue			
Low	30 to 43	56	50.5
High	44 to 95	55	49.5

High fatigue was significantly associated (p < 0.05) with the following variables: marital status, educational level, alcohol consumption, feeling upon waking, sleeping in environments with external noise, sleep quality, operating vehicles at work, satisfaction with salary, satisfaction with career development opportunities, and the impact of work on personal life (Table 2).

**Table 2:** Crude odds ratio (OR) for high fatigue among mining workers (n = 111), according to biosocial characteristics and work-related factors, Poços de Caldas, Minas Gerais, Brazil, 2023

Fatigue dimensions and overall classification	Dimensions of the stress scale based on the demand-control-social support model											
	Demand				Control				Social support			
	Low (n = 63)		High (n = 48)		Low (n = 69)		High (n = 42)		Low (n = 60)		High (n = 51)	
	n	(%)	n	(%)	n	(%)	n	(%)	n	(%)	n	(%)
Sleepiness and unwillingness to work												
Low	42	66.7	18	37.5	42	60.9	18	42.9	36	60.0	20	39.2
High	21	33.3	30	62.5	27	39.1	24	57.1	24	40.0	31	60.8
Difficulty with concentration and attention												
Low	41	65.1	22	45.8	45	65.2	18	42.9	40	66.7	20	39.2
High	22	34.9	26	54.2	24	34.8	24	57.1	20	33.3	31	60.8
Physical discomfort												
Low	44	69.8	24	50.0	43	62.3	25	59.5	38	63.3	25	49.0
High	19	30.2	24	50.0	26	37.7	17	40.5	22	36.7	26	51.0
Overall fatigue												
Low	19	30.2	37	77.1	39	56.5	17	40.5	38	63.3	25	49.0
High	29	46.0	26	54.2	30	43.5	25	59.5	22	36.7	26	51.0

Biosocial variables and work-related factors associated with each dimension of the fatigue assessment scale, along with their respective crude ORs, are presented in Table 3.

**Table 3:** Crude odds ratio (OR) for sleepiness and unwillingness to work, difficulty with concentration and attention, and physical discomfort among mining workers (n = 111), according to biosocial characteristics and work-related factors, Poços de Caldas, Minas Gerais, Brazil, 2023

Variables	Category	p-value	Crude OR (95% CI)
Marital status	Married vs. single	0.01^*^	3.37 (1.44-7.74)
Education	Primary vs. higher education	0.02^*^	3.31 (1.11-9.82)
Alcohol consumption	Yes vs. no	0.01^*^	2.90 (1.30-6.43)
Feeling upon waking	Slightly tired vs. well rested	< 0.01^*^	5.24 (1.78-15.45)
External noise during sleep	Yes vs. no	< 0.01†	4.87 (1.50-15.82)
			
Sleep quality	Poor or fair vs. excellent	< 0.01†	10.08 (2.41-42.04)
	Good vs. excellent		5.32 (1.79-15.76)
			
Driving vehicles at work	No vs. yes	< 0.01†	5.02 (2.05-12.29)
Salary satisfaction	Dissatisfied vs. very satisfied	0.01†	8.00 (1.60-39.96)
Career development opportunities	Dissatisfied vs. very satisfied	0.04†	5.85 (1.22-27.99)
			
Impact of work on personal life	Slight impact vs. no impact	< 0.01^*^	2.77 (1.15-6.67)
	Moderate impact vs. no impact		7.49 (1.49-37.65)

* Fisher’s exact test.

† Chi-square test.

High psychological demand was associated with all dimensions of fatigue (sleepiness and unwillingness to work, difficulty with concentration and attention, and physical discomfort), whereas low control was associated with sleepiness and unwillingness to work and difficulty with concentration and attention, and low social support was associated with sleepiness and unwillingness to work and physical discomfort (Table 4).

**Table 4:** Crude odds ratio (OR) for the dimensions of the fatigue assessment scale and the dimensions of the work stress scale among mining workers (n = 111), Poços de Caldas, Minas Gerais, Brazil, 2023

Fatigue dimension	Variables	Category	p-value	Crude OR (95% CI)
Sleepiness and unwillingness to work	Education	Primary vs. higher education	0.04^*^	5.85 (1.22-27.99)
	Feeling upon waking	Slightly tired vs. well rested	< 0.01^*^	8.86 (2.76-28.44)
	Sleep quality	Poor/fair vs. excellent	< 0.01†	13.19 (2.97-58.61)
		Good vs. excellent		5.83 (1.81-18.72)
	Work interference with sleep	Yes vs. no	0.01^*^	6.21 (1.27-30.25)
	Driving vehicles at work	No vs. yes	< 0.01†	3.35 (1.51-8.24)
	Salary satisfaction	Dissatisfied vs. very satisfied	0.03†	8.74 (1.52-50.29)
	Career development opportunities	Dissatisfied vs. very satisfied	0.02†	5.85 (1.22-27.99)
	Meal satisfaction	Dissatisfied vs. very satisfied	< 0.01†	21.00 (2.15-204.61)
				
Difficulty with concentration and attention	Marital status	Married vs. single	0.02†	2.93 (1.27-6.71)
	Education	Primary vs. higher education	0.04^*^	6.66 (1.46-30.42)
	Place of sleep	Dormitory vs. own home	0.02^*^	5.90 (1.19-29.24)
	Melatonin use for sleep	Yes vs. no	< 0.01^*^	7.17 (1.36-37.80)
	Feeling upon waking	Slightly tired vs. well rested	< 0.01^*^	4.26 (1.58-11.51)
	External noise during sleep	Yes vs. no	< 0.01^*^	4.77 (1.58-14.42)
	Sleep quality	Poor vs. excellent	0.01^*^	12.60 (1.07-148.12)
		Fair vs. excellent		6.72 (1.52-29.61)
		Good vs. excellent		3.73 (1.26-11.05)
	Work interference with sleep	Yes vs. no	< 0.01^*^	7.03 (1.44-34.30)
	Driving vehicles at work	No vs. yes	< 0.01†	2.94 (1.28-6.74)
	Work’s impact on personal life	Moderate vs. no impact	0.01^*^	9.21 (1.82-46.49)
				
Physical discomfort	Education	Primary vs. higher education	0.02^*^	9.52 (2.01-44.91)
	Alcohol consumption	Yes vs. no	0.02^*^	2.78 (1.24-6.25)
	Feeling upon waking	Slightly tired vs. well rested	0.04†	2.25 (1.00-6.52)
	External noise during sleep	Yes vs. no	0.01†	3.37 (1.20-9.42)
	Sleep quality	Poor vs. excellent	< 0.01^*^	23.00 (1.77-298.44)
	Driving vehicles at work	No vs. yes	< 0.01^*^	4.56 (1.94-10.75)
	Salary satisfaction	Dissatisfied vs. very satisfied	0.01†	8.74 (1.52-50.30)
	Work’s impact on personal life	Slight/moderate vs. no impact	0.02^*^	3.42 (1.54-7.61)

* Fisher’s exact test.

† Chi-square test.

The statistical and comparative analysis of the fatigue and work stress scales between job functions (being a heavy vehicle driver vs. not being a heavy vehicle driver) showed that non-drivers had a higher likelihood of experiencing high fatigue (OR = 4.68; 95% CI 1.92-11.38), sleepiness and unwillingness to work (OR = 3.28; 95% CI 1.41-7.60), difficulty with concentration and attention (OR = 2.72; 95% CI 1.19-6.20), and greater physical discomfort (OR = 4.43; 95% CI 1.89-;10.37). In addition, non-drivers were also more likely to be exposed to high psychological demands at work (OR = 2.28; 95% CI 1.01-5.16).

## DISCUSSION

Fatigue is a significant concern in the field of occupational health and safety.^10^ Characterized as a complex wear-and-tear process triggered by biopsychosocial factors, its effects impact physical, cognitive, emotional, and even social health, reducing work capacity and productivity while increasing the risk of occupational accidents.^11,12^

Workplace fatigue can also be measured using technological tools (eg, assessment of eye and head movements, heart rate, and psychomotor vigilance) or self-reported instruments.^13,14^ While technology aims to indirectly assess the physiological effects of fatigue, self-report questionnaires seek to capture individuals’ perceptions of tiredness or lack of energy.

Mining work is diverse, and day-to-day operations vary depending on the sector, excavation techniques, equipment and vehicles used, and the mine’s characteristics. The wide range of tasks, lack of training, ergonomic challenges, exposure to heat, noise, and vibration, monotonous work, remote job sites, isolation, work-life imbalance, and overall workload make these workers more susceptible to fatigue compared to those in other industrial sectors.^15^

Although frequently linked to sleep deprivation, fatigue is also associated with living conditions and job characteristics such as shift schedule, job function, work demands, working hours, rest time, and the degree of social support from supervisors and coworkers.^16^

Studies show that individuals’ perceptions of their health status and the importance of healthy lifestyle habits are influenced by educational level, which may increase vulnerability to illness due to limited access to information, opportunities, and preventive resources.^10,17^ In this study, low educational attainment was associated with high fatigue and all of its dimensions (sleepiness and unwillingness to work, difficulty with concentration and attention, and physical discomfort).

Married workers or those in stable unions were at greater risk of fatigue and more likely to report difficulty concentrating and paying attention. This association may be explained by the additional burden of family responsibilities, household tasks, and factors that interfere with sleep - such as bedtime routines, the presence of infants, and the partner’s snoring - as well as the increased potential for work-family conflict.^18^

Job characteristics in the mining sector often disrupt the balance between personal life and work. Experiencing such imbalance contributes to symptoms of psychological exhaustion and strain.^19^ Similar to findings from a study on railroad workers,^19^ our results showed that the greater the impact of work on personal life, the higher the risk of severe fatigue. Moreover, work-life conflict was also associated with difficulty concentrating and with physical discomfort, since workers experiencing these conflicts are more likely to exhibit signs of psychological exhaustion and strain.^19^

Regarding lifestyle habits, this study found that alcohol consumption tripled the risk of fatigue. The relationship between fatigue and alcohol is complex: although alcohol may initially help reduce feelings of tiredness, it ultimately increases fatigue due to its effects on sleep quality, physical discomfort, reduced alertness, and cognitive impairment.^14,20^

Tasks that require long-distance travel, remote work, and sleeping in shared accommodations tend to be more physically and mentally exhausting.^21,22^ Sleeping in dormitories increases fatigue and concentration difficulties due to poorer sleep quality, often caused by noise exposure and reduced comfort.^23^

The relationship between sleep and workplace fatigue is one of the most frequently studied, particularly in shift work contexts. In this study, work shift was not associated with worker fatigue, and overall, most participants did not report sleepiness or unwillingness to work. These findings may be explained by the fact that fatigue related to shift work depends on the timing and duration of the shift. Furthermore, although the tasks were relatively exhausting and monotonous, the intershift rest periods and scheduled days off may have been effective in relieving fatigue.^24^

Sleep is invariably the primary strategy for fatigue recovery, and evaluating it requires consideration of 2 factors: sleep quality and sleep duration. The former refers to aspects that affect the restorative capacity of sleep and how the person feels upon waking, while the latter concerns the number of hours slept. Both are strongly correlated with fatigue.^14,20,21,25^

Sleep quality is closely linked to well-being, anxiety, tension, work and family conflicts, and job satisfaction. Mining workers tend to experience poorer sleep quality, even when sleeping a sufficient number of hours.^15^ This may be due to external factors or work routines that prevent proper recovery, as evidenced in this study.

Developing strategies to promote rest and recovery - especially outside working hours, such as improving accommodations - may help mitigate the effects of fatigue in mining operations.^15^

Melatonin is commonly used to improve sleep quality. However, inappropriate dosages or timing of melatonin supplementation can result in fatigue, sleepiness, and reduced productivity and concentration,^26^ as observed in this study.

Job satisfaction in specific areas was also associated with fatigue. This may be because satisfaction with salary and job stability are considered protective factors against fatigue, boredom, lack of motivation, and low productivity.^11,12,21^

Workplace fatigue may result not only from mentally or physically exhausting tasks but also from monotonous work and low job autonomy.^5,11,22^ Although driving heavy vehicles in mining settings tends to increase fatigue - due to the repetitive nature of the job, following the same route daily at very low speeds^13^ - this study found that higher fatigue prevalence was observed among workers in administrative roles or those operating only mobile equipment or light vehicles.

Overall, most workers in this mining company were exposed to passive work conditions, defined by both low demand and low control/autonomy. This type of work is considered the second most harmful, as it is associated with boredom and a lack of challenges, which may lead to disinterest, skill loss, and decreased productivity.^27^

Additionally, no association was found between job type and fatigue. However, these findings do not rule out a possible link between being a driver and experiencing fatigue, since many of the most fatigued individuals in the sample were those who operated mobile equipment or light vehicles under passive work conditions.

When psychosocial factors at work were analyzed separately, high demand was found to be associated with all dimensions of fatigue. Work demands are related to job performance and can be understood as the result of the interaction between workers’ perceptions and the requirements, skills, behaviors, and environment involved in their work. An imbalance between the physical, psychological, social, and organizational demands of the work environment and the resources available to meet goals or objectives (eg, sleep and rest, nutrition, autonomy, social support) can lead to illness and exhaustion.^5,28^

One mitigating resource against occupational demands, mental exhaustion, stress, and fatigue is social support. A study found that higher levels of supervisory support were associated with lower fatigue, less physical effort, greater motivation, and reduced work-family conflict.^19^

In this study, it was observed that in addition to exposure to passive work, most workers reported low social support. This was associated with high levels of fatigue, sleepiness and unwillingness to work, and physical discomfort. Lack of support and abusive behaviors by supervisors - even when unintentional - can result in physical and psychological harm, such as sleep disturbances, fatigue, difficulty concentrating, stress, anxiety, and emotional exhaustion. Furthermore, the negative effects of a lack of leadership support may spill over into workers’ personal lives, as frustration and tension are often transferred to family members.^29^

No occupation is immune to fatigue; therefore, it is not possible to standardize or exhaustively list all fatigue-related factors in every work environment and profession. The complexity of this issue requires fatigue to be assessed through an alternative approach that includes identifying tasks most susceptible to fatigue, the risk of error as fatigue sets in during each activity, and which measures are most effective in preventing or reducing fatigue in that specific context.^30^

Prior to this study, the company already employed several daily fatigue monitoring and screening strategies, including an online neuropsychological test, fatigue-detection cameras installed in heavy vehicle cabins, and regular safety dialogues that encouraged workers to report signs or symptoms of fatigue before beginning their shifts. Therefore, it is possible that both the psychosocial factors associated with fatigue and the overall prevalence of fatigue in this population may have been mitigated by these strategies.

Moreover, as this is a cross-sectional study, it is not possible to confirm causality between the identified associations and fatigue, even though the results are consistent with those reported in the literature in other occupational contexts. Another limitation lies in the potential “healthy worker effect” (self-selection bias), which is common in workplace research, since the most fatigued individuals may have been absent from work due to vacation, medical leave, or other reasons.

Nonetheless, the relevance of this study lies in the fact that fatigue in mining remains an underexplored topic. Additionally, its originality stems from examining the relationship between psychosocial factors based on the demand-control model and fatigue in this specific occupational setting.

## CONCLUSIONS

This study rejected the hypothesis that driving heavy vehicles increases fatigue in mining work and confirmed that sleep and rest-related factors are associated with and increase the risk of fatigue - especially external noise in the sleeping environment and the perception of poor sleep quality.

Work demand was the psychosocial factor most strongly associated with all dimensions of fatigue. Moreover, the findings showed that passive work can be just as fatiguing as high-strain work, and low social support appears to be an aggravating factor in such situations. These findings highlight the importance of future studies on the evaluation of work-related stress and its relationship with fatigue, based on the demand-control and social support model.
