# Erratum: Psychosocial factors associated with fatigue in mining work

**DOI:** 10.47626/10.47626/1679-4435-2025-1492Erratum

**Published:** 2026-04-15

This erratum corrects: http://doi.org/10.47626/1679-4435-2025-1492

The authors of the article titled “Psychosocial factors associated with fatigue in mining work” (http://doi.org/10.47626/1679-4435-2025-1492), published in its final form in volume 23 issue 3 (2025), reported an error in the ordering of the article’s tables, which involved the removal of one table and the inclusion of another table. To resolve the inconsistency, Table 1 was removed and a new Table 4 was included.

**Table 1:** Distribution of workers (n = 111) according to dimensions of stress and fatigue assessment scales, Poços de Caldas, Minas Gerais, Brazil, 2023

Dimension	Cutoff points	n	(%)
Sleepiness and unwillingness to work			
Low	10 to 14	60	54.1
High	15 to 36	51	45.9
Difficulty with concentration and attention			
Low	10 to 17	63	56.8
High	18 to 41	48	43.2
Physical discomfort			
Low	10 to 12	68	61.3
High	13 to 30	43	38.7
Overall fatigue			
Low	30 to 43	56	50.5
High	44 to 95	55	49.5

**Table 2:** Crude odds ratio (OR) for high fatigue among mining workers (n = 111), according to biosocial characteristics and work-related factors, Poços de Caldas, Minas Gerais, Brazil, 2023

Fatigue dimensions and overall classification	Dimensions of the stress scale based on the demand-control-social support model											
	Demand				Control				Social support			
	Low (n = 63)		High (n = 48)		Low (n = 69)		High (n = 42)		Low (n = 60)		High (n = 51)	
	n	(%)	n	(%)	n	(%)	n	(%)	n	(%)	n	(%)
Sleepiness and unwillingness to work												
Low	42	66.7	18	37.5	42	60.9	18	42.9	36	60.0	20	39.2
High	21	33.3	30	62.5	27	39.1	24	57.1	24	40.0	31	60.8
Difficulty with concentration and attention												
Low	41	65.1	22	45.8	45	65.2	18	42.9	40	66.7	20	39.2
High	22	34.9	26	54.2	24	34.8	24	57.1	20	33.3	31	60.8
Physical discomfort												
Low	44	69.8	24	50.0	43	62.3	25	59.5	38	63.3	25	49.0
High	19	30.2	24	50.0	26	37.7	17	40.5	22	36.7	26	51.0
Overall fatigue												
Low	19	30.2	37	77.1	39	56.5	17	40.5	38	63.3	25	49.0
High	29	46.0	26	54.2	30	43.5	25	59.5	22	36.7	26	51.0

**Table 3:** Crude odds ratio (OR) for sleepiness and unwillingness to work, difficulty with concentration and attention, and physical discomfort among mining workers (n = 111), according to biosocial characteristics and work-related factors, Poços de Caldas, Minas Gerais, Brazil, 2023

Variables	Category	p-value	Crude OR (95% CI)
Marital status	Married vs. single	0.01^*^	3.37 (1.44-7.74)
Education	Primary vs. higher education	0.02^*^	3.31 (1.11-9.82)
Alcohol consumption	Yes vs. no	0.01^*^	2.90 (1.30-6.43)
Feeling upon waking	Slightly tired vs. well rested	< 0.01^*^	5.24 (1.78-15.45)
External noise during sleep	Yes vs. no	< 0.01^†^	4.87 (1.50-15.82)
			
Sleep quality	Poor or fair vs. excellent	< 0.01^†^	10.08 (2.41-42.04)
	Good vs. excellent		5.32 (1.79-15.76)
			
Driving vehicles at work	No vs. yes	< 0.01^†^	5.02 (2.05-12.29)
Salary satisfaction	Dissatisfied vs. very satisfied	0.01^†^	8.00 (1.60-39.96)
Career development opportunities	Dissatisfied vs. very satisfied	0.04^†^	5.85 (1.22-27.99)
			
Impact of work on personal life	Slight impact vs. no impact	< 0.01^*^	2.77 (1.15-6.67)
	Moderate impact vs. no impact		7.49 (1.49-37.65)

* Fisher’s exact test.

† Chi-square test.

**Table 4:** Crude odds ratio (OR) for the dimensions of the fatigue assessment scale and the dimensions of the work stress scale among mining workers (n = 111), Poços de Caldas, Minas Gerais, Brazil, 2023

Fatigue dimension	Variables	Category	p-value	Crude OR (95% CI)
Sleepiness and unwillingness to work	Education	Primary vs. higher education	0.04^*^	5.85 (1.22-27.99)
	Feeling upon waking	Slightly tired vs. well rested	< 0.01^*^	8.86 (2.76-28.44)
	Sleep quality	Poor/fair vs. excellent	< 0.01^†^	13.19 (2.97-58.61)
		Good vs. excellent		5.83 (1.81-18.72)
	Work interference with sleep	Yes vs. no	0.01^*^	6.21 (1.27-30.25)
	Driving vehicles at work	No vs. yes	< 0.01^†^	3.35 (1.51-8.24)
	Salary satisfaction	Dissatisfied vs. very satisfied	0.03^†^	8.74 (1.52-50.29)
	Career development opportunities	Dissatisfied vs. very satisfied	0.02^†^	5.85 (1.22-27.99)
	Meal satisfaction	Dissatisfied vs. very satisfied	< 0.01^†^	21.00 (2.15-204.61)
				
Difficulty with concentration and attention	Marital status	Married vs. single	0.02^†^	2.93 (1.27-6.71)
	Education	Primary vs. higher education	0.04^*^	6.66 (1.46-30.42)
	Place of sleep	Dormitory vs. own home	0.02^*^	5.90 (1.19-29.24)
	Melatonin use for sleep	Yes vs. no	< 0.01^*^	7.17 (1.36-37.80)
	Feeling upon waking	Slightly tired vs. well rested	< 0.01^*^	4.26 (1.58-11.51)
	External noise during sleep	Yes vs. no	< 0.01^*^	4.77 (1.58-14.42)
	Sleep quality	Poor vs. excellent	0.01^*^	12.60 (1.07-148.12)
		Fair vs. excellent		6.72 (1.52-29.61)
		Good vs. excellent		3.73 (1.26-11.05)
	Work interference with sleep	Yes vs. no	< 0.01^*^	7.03 (1.44-34.30)
	Driving vehicles at work	No vs. yes	< 0.01^†^	2.94 (1.28-6.74)
	Work’s impact on personal life	Moderate vs. no impact	0.01^*^	9.21 (1.82-46.49)
				
Physical discomfort	Education	Primary vs. higher education	0.02^*^	9.52 (2.01-44.91)
	Alcohol consumption	Yes vs. no	0.02^*^	2.78 (1.24-6.25)
	Feeling upon waking	Slightly tired vs. well rested	0.04^†^	2.25 (1.00-6.52)
	External noise during sleep	Yes vs. no	0.01^†^	3.37 (1.20-9.42)
	Sleep quality	Poor vs. excellent	< 0.01^*^	23.00 (1.77-298.44)
	Driving vehicles at work	No vs. yes	< 0.01^*^	4.56 (1.94-10.75)
	Salary satisfaction	Dissatisfied vs. very satisfied	0.01^†^	8.74 (1.52-50.30)
	Work’s impact on personal life	Slight/moderate vs. no impact	0.02^*^	3.42 (1.54-7.61)

* Fisher’s exact test.

† Chi-square test.

**Table 1:** Distribution of workers (n = 111) according to dimensions of the stress and fatigue assessment scales, Poços de Caldas, MG, Brazil, 2023

Fatigue dimensions and overall classification	Dimensions of the stress scale based on the demand-control-social support model											
	Demand				Control				Social support			
	Low (n = 63)		High (n = 48)		Low (n = 69)		High (n = 42)		Low (n = 60)		High (n = 51)	
	n	(%)	n	(%)	n	(%)	n	(%)	n	(%)	n	(%)
Sleepiness and unwillingness to work												
Low	42	66.7	18	37.5	42	60.9	18	42.9	36	60.0	20	39.2
High	21	33.3	30	62.5	27	39.1	24	57.1	24	40.0	31	60.8
Difficulty with concentration and attention												
Low	41	65.1	22	45.8	45	65.2	18	42.9	40	66.7	20	39.2
High	22	34.9	26	54.2	24	34.8	24	57.1	20	33.3	31	60.8
Physical discomfort												
Low	44	69.8	24	50.0	43	62.3	25	59.5	38	63.3	25	49.0
High	19	30.2	24	50.0	26	37.7	17	40.5	22	36.7	26	51.0
Overall fatigue												
Low	19	30.2	37	77.1	39	56.5	17	40.5	38	63.3	25	49.0
High	29	46.0	26	54.2	30	43.5	25	59.5	22	36.7	26	51.0

**Table 2:** Crude odds ratio (OR) for high fatigue among mining workers (n = 111), according to biosocial characteristics and work-related factors, Poços de Caldas, Minas Gerais, Brazil, 2023

Variables	Category	p-value	Crude OR (95% CI)
Marital status	Married vs. single	0.01^*^	3.37 (1.44-7.74)
Education	Primary vs. higher education	0.02^*^	3.31 (1.11-9.82)
Alcohol consumption	Yes vs. no	0.01^*^	2.90 (1.30-6.43)
Feeling upon waking	Slightly tired vs. well rested	< 0.01^*^	5.24 (1.78-15.45)
External noise during sleep	Yes vs. no	< 0.01^†^	4.87 (1.50-15.82)
Sleep quality	Poor or fair vs. excellent	< 0.01^†^	10.08 (2.41-42.04)
	Good vs. excellent		5.32 (1.79-15.76)
Driving vehicles at work	No vs. yes	< 0.01^†^	5.02 (2.05-12.29)
Salary satisfaction	Dissatisfied vs. very satisfied	0.01^†^	8.00 (1.60-39.96)
Career development opportunities	Dissatisfied vs. very satisfied	0.04^†^	5.85 (1.22-27.99)
Impact of work on personal life	Slight impact vs. no impact	< 0.01^*^	2.77 (1.15-6.67)
	Moderate impact vs. no impact		7.49 (1.49-37.65)

* Fisher’s exact test.

† Chi-square test.

**Table 3:** Crude odds ratio (OR) for sleepiness and unwillingness to work, difficulty with concentration and attention, and physical discomfort among mining workers (n = 111), according to biosocial characteristics and work-related factors, Poços de Caldas, Minas Gerais, Brazil, 2023

Fatigue dimension	Variables	Category	p-value	Crude OR (95% CI)
^*^ Fisher’s exact test.	Education	Primary vs. higher education	0.04^*^	5.85 (1.22-27.99)
	^†^ Chi-square test.	Slightly tired vs. well rested	< 0.01^*^	8.86 (2.76-28.44)
	Sleep quality	Poor/fair vs. excellent	< 0.01^†^	13.19 (2.97-58.61)
		Good vs. excellent		5.83 (1.81-18.72)
	Work interference with sleep	Yes vs. no	0.01^*^	6.21 (1.27-30.25)
	Driving vehicles at work	No vs. yes	< 0.01^†^	3.35 (1.51-8.24)
	Salary satisfaction	Dissatisfied vs. very satisfied	0.03^†^	8.74 (1.52-50.29)
	Career development opportunities	Dissatisfied vs. very satisfied	0.02^†^	5.85 (1.22-27.99)
	Meal satisfaction	Dissatisfied vs. very satisfied	< 0.01^†^	21.00 (2.15-204.61)
Difficulty with concentration and attention	Marital status	Married vs. single	0.02^†^	2.93 (1.27-6.71)
	Education	Primary vs. higher education	0.04^*^	6.66 (1.46-30.42)
	Place of sleep	Dormitory vs. own home	0.02^*^	5.90 (1.19-29.24)
	Melatonin use for sleep	Yes vs. no	< 0.01^*^	7.17 (1.36-37.80)
	Feeling upon waking	Slightly tired vs. well rested	< 0.01^*^	4.26 (1.58-11.51)
	External noise during sleep	Yes vs. no	< 0.01^*^	4.77 (1.58-14.42)
	Sleep quality	Poor vs. excellent	0.01^*^	12.60 (1.07-148.12)
		Fair vs. excellent		6.72 (1.52-29.61)
		Good vs. excellent		3.73 (1.26-11.05)
	Work interference with sleep	Yes vs. no	< 0.01^*^	7.03 (1.44-34.30)
	Driving vehicles at work	No vs. yes	< 0.01^†^	2.94 (1.28-6.74)
	Work’s impact on personal life	Moderate vs. no impact	0.01^*^	9.21 (1.82-46.49)
Physical discomfort	Education	Primary vs. higher education	0.02^*^	9.52 (2.01-44.91)
	Alcohol consumption	Yes vs. no	0.02^*^	2.78 (1.24-6.25)
	Feeling upon waking	Slightly tired vs. well rested	0.04^†^	2.25 (1.00-6.52)
	External noise during sleep	Yes vs. no	0.01^†^	3.37 (1.20-9.42)
	Sleep quality	Poor vs. excellent	< 0.01^*^	23.00 (1.77-298.44)
	Driving vehicles at work	No vs. yes	< 0.01^*^	4.56 (1.94-10.75)
	Salary satisfaction	Dissatisfied vs. very satisfied	0.01^†^	8.74 (1.52-50.30)
	Work’s impact on personal life	Slight/moderate vs. no impact	0.02^*^	3.42 (1.54-7.61)

* Fisher’s exact test.

† Chi-square test.

**Table 4:** Crude odds ratio (OR) for the dimensions of the fatigue assessment scale and the dimensions of the work stress scale among mining workers (n = 111). Poços de Caldas, MG, Brazil, 2023

Dimension	p	Crude OR (95% CI)
Sleepiness and unwillingness to work		
Demand	0.02^*^	3.33 (1.52-7.30)
Control	0.06^*^	1.35 (0.81-4.67)
Social support	<0.01^*^	3.10 (1.42-6.74)
Difficulty with concentration and attention		
Demand	0.04^*^	2.20 (1.02-4.75)
Control	0.02^*^	2.50 (1.18-5.49)
Social support	0.12^*^	1.12 (0.63-3.62)
Physical discomfort		
Demand	0.03^*^	2.31 (1.06-5.05)
Control	0.76^*^	1.21 (0.79-4.67)
Social support	0.04^*^	2.24 (1.03-4.88)

* Chi-square test.

Where it read:

It should read:

Approved by Sergio Roberto de Lucca, Editor-in-Chief.

